# Inhibiting membrane rupture with NINJ1 antibodies limits tissue injury

**DOI:** 10.1038/s41586-023-06191-5

**Published:** 2023-05-17

**Authors:** Nobuhiko Kayagaki, Irma B. Stowe, Kamela Alegre, Ishan Deshpande, Shuang Wu, Zhonghua Lin, Opher S. Kornfeld, Bettina L. Lee, Juan Zhang, John Liu, Eric Suto, Wyne P. Lee, Kellen Schneider, WeiYu Lin, Dhaya Seshasayee, Tushar Bhangale, Cecile Chalouni, Matthew C. Johnson, Prajakta Joshi, Jan Mossemann, Sarah Zhao, Danish Ali, Neil M. Goldenberg, Blayne A. Sayed, Benjamin E. Steinberg, Kim Newton, Joshua D. Webster, Ryan L. Kelly, Vishva M. Dixit

**Affiliations:** 1 Department of Physiological Chemistry, Genentech, South San Francisco, CA USA; 2 Department of Structural Biology, Genentech, South San Francisco, CA USA; 3 Department of Antibody Engineering, Genentech, South San Francisco, CA USA; 4 Department of Translational Immunology, Genentech, South San Francisco, CA USA; 5 Department of Human Genetics, Genentech, South San Francisco, CA USA; 6 Department of Pathology, Genentech, South San Francisco, CA USA; 7 Department of Biomolecular Resources, Genentech, South San Francisco, CA USA; 8 Program in Cell Biology, Hospital for Sick Children, Toronto, Ontario Canada; 9 Program in Neuroscience and Mental Health, Hospital for Sick Children, Toronto, Ontario Canada; 10 Department of Anesthesia and Pain Medicine, Hospital for Sick Children, Toronto, Ontario Canada; 11 Division of General Surgery, Hospital for Sick Children, Toronto, Ontario Canada

**Keywords:** Immune cell death, Apoptosis

## Abstract

Plasma membrane rupture (PMR) in dying cells undergoing pyroptosis or apoptosis requires the cell-surface protein NINJ1^1^. PMR releases pro-inflammatory cytoplasmic molecules, collectively called damage-associated molecular patterns (DAMPs), that activate immune cells. Therefore, inhibiting NINJ1 and PMR may limit the inflammation that is associated with excessive cell death. Here we describe an anti-NINJ1 monoclonal antibody that specifically targets mouse NINJ1 and blocks oligomerization of NINJ1, preventing PMR. Electron microscopy studies showed that this antibody prevents NINJ1 from forming oligomeric filaments. In mice, inhibition of NINJ1 or *Ninj1* deficiency ameliorated hepatocellular PMR induced with TNF plus d-galactosamine, concanavalin A, Jo2 anti-Fas agonist antibody or ischaemia–reperfusion injury. Accordingly, serum levels of lactate dehydrogenase, the liver enzymes alanine aminotransaminase and aspartate aminotransferase, and the DAMPs interleukin 18 and HMGB1 were reduced. Moreover, in the liver ischaemia–reperfusion injury model, there was an attendant reduction in neutrophil infiltration. These data indicate that NINJ1 mediates PMR and inflammation in diseases driven by aberrant hepatocellular death.

## Abstract (web summary)

A monoclonal antibody that binds NINJ1 and inhibits NINJ1 oligomerization prevents plasma membrane rupture in dying cells, resulting in decreased inflammation of surrounding tissue in mice.

## Main

NINJ1 is a 16 kDa cell-surface protein predicted to have two transmembrane regions and both N and C termini on the outside of the cell^2,3^. Although it is dispensable for the induction of cell death, NINJ1 controls an important consequence of apoptotic or pyroptotic cell death, mediating PMR that non-selectively releases pro-inflammatory cytoplasmic contents from dying cells^1,4^. Whether NINJ1-dependent PMR exacerbates tissue damage in disease is unclear, but *Ninj1* deficiency is reported to attenuate mouse models of pulmonary fibrosis and multiple sclerosis^5,6^. Given that a conserved extracellular region of NINJ1 is essential for its oligomerization and PMR^1^, we hypothesized that extracellular anti-NINJ1 antibodies could be used to inhibit NINJ1-dependent PMR in vivo.

## Identifying NINJ1-blocking antibodies

**Fig. 1:**
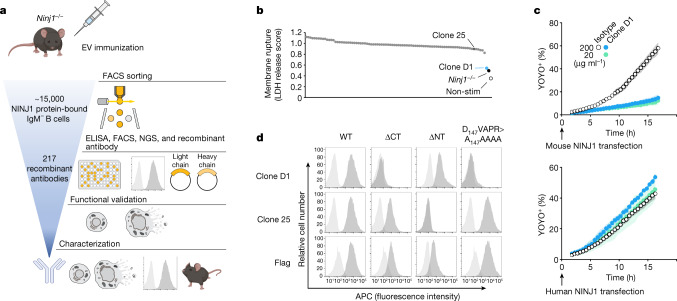
Identification of NINJ1-blocking antibody clone D1. **a**, Scheme of recombinant antibody screening. EV, extracellular vesicle; FACS, fluorescence-activated cell sorting. **b**, LDH released from Pam3CSK4-primed wild-type BMDMs after nigericin stimulation for 16 h in the presence of 1 μg ml^−1^ of indicated antibody (dots represent different antibodies tested). *Ninj1*^−/−^, *Ninj1*^−/−^ BMDMs; non-stim, non-stimulated wild-type BMDMs. The LDH score is the LDH release normalized against the no-antibody control. **c**, The percentage of YOYO-1^+^ NINJ1-expressing HEK293T cells when cultured with clone D1 or an isotype control antibody. Data are mean (circles) ± s.d. (shaded area) of three independent replicates. **d**, Flow cytometry histograms of propidium iodide-negative HEK293T cells surface-stained with anti-NINJ1 or anti-Flag antibodies. Cells are mock-transfected (light grey) or transfected with indicated NINJ1 constructs (dark grey). WT, wild type; ΔCT, NINJ1(Δ142–152); ΔNT, NINJ1(Δ2–73). In **c**,**d**, results are representative of three independent experiments.

**Extended Data Fig. 1:**
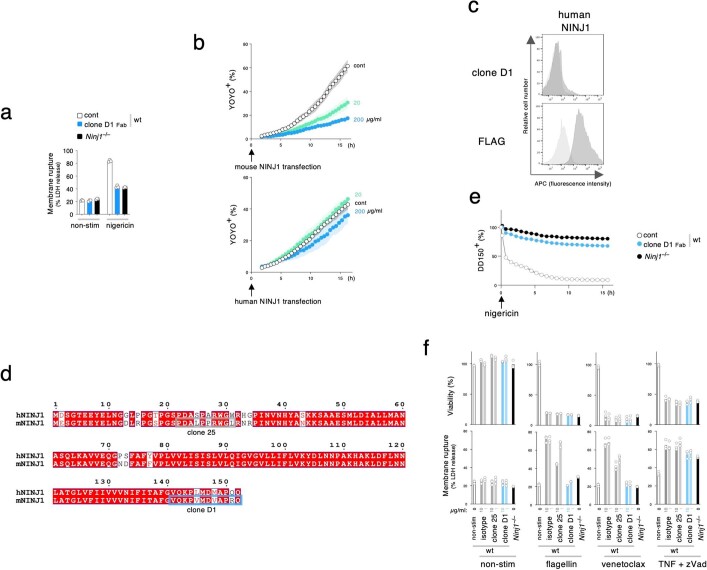
Clone D1 inhibits PMR. **a**, Graphs indicate LDH release in cultures of wild-type BMDMs after priming, and then stimulation with nigericin for 16 h in the presence or absence (cont) of clone D1 Fab. Non-stim, non-stimulated control. Bars indicate the mean. Circles indicate 3 biological replicates. Results representative of 3 independent experiments. **b**, Graphs indicate the percentage of YOYO-1^+^ HEK293T NINJ1 expressing cells when cultured with or without (cont) clone D1 Fab. Circles are the mean ± s.d. (shaded area) of 3 independent replicates. Results are representative of 2 independent experiments. **c**, Flow cytometry histograms of propidium iodide-negative HEK293T cells surface-stained with anti-NINJ1 or anti-FLAG antibodies. Cells are mock transfected (light gray) or transfected with human NINJ1 (dark gray). Results representative of 3 independent experiments. **d**, Amino acid sequence alignment of human and mouse NINJ1. Predicted epitope of clone 25 (grey box) and clone D1 (blue box) are highlighted. **e**, Graph shows the release of DD-150 from Pam3CSK4-primed BMDMs in (**a**) after nigericin stimulation. Data are means (circles) ± s.d. (shaded area). Circles indicate 3 biological replicates (*n* = 3 animals). Results representative of 3 independent experiments. **f**, Graphs indicate cell viability (top) and LDH release (bottom) in BMDMs. Non-stim, non-stimulated control. Bars indicate the mean. Circles indicate 3 biological replicates (*n* = 3 animals). Results representative of 2 independent experiments.

To generate NINJ1-blocking antibodies, we immunized *Ninj1*^−/−^ mice with extracellular vesicles expressing full-length mouse NINJ1 (Fig. 1a). We isolated approximately 15,000 NINJ1-binding IgM^−^ B cells by flow cytometric analysis and characterized single-cell supernatants for binding to NINJ1-expressing cells. Functional screening of 217 recombinant anti-mouse NINJ1 IgG2a monoclonal antibodies identified clone D1 (Ninj1–575) as an inhibitor of NINJ1-dependent PMR (Fig. 1b). Mouse bone marrow-derived macrophages (BMDMs) were primed with the Toll-like receptor 2 (TLR2) agonist Pam3CSK4 to up-regulate inflammasome components, including *Nlrp3*, and then cultured with 1 µg ml^−1^ anti-NINJ1 antibodies for 15 min prior to stimulation with nigericin to activate NLRP3- and GSDMD-dependent pyroptosis^7,8^. Lactate dehydrogenase (LDH) release was used to monitor NINJ1-dependent PMR^1^. The most potent anti-NINJ1 antagonist antibody, clone D1, reduced PMR in wild-type BMDMs to levels observed in *Ninj1*^−/−^ control BMDMs. The antigen-binding fragment (Fab) of clone D1 also prevented nigericin-induced PMR in wild-type BMDMs, suggesting that clone D1 can inhibit NINJ1 independently of binding to Fc receptors on the cell surface (Extended Data Fig. 1a). Clone D1 or its Fab also suppressed membrane damage caused by ectopic expression of mouse NINJ1, but not human NINJ1, in HEK293T cells (Fig. 1c and Extended Data Fig. 1b).

We confirmed that clone D1 recognized mouse NINJ1, but not human NINJ1, by surface staining and flow cytometric analysis of live HEK293T cells expressing N-terminally Flag-tagged mouse NINJ1 (Fig. 1d) or Flag-tagged human NINJ1 (Extended Data Fig. 1c). Antibody binding to mouse NINJ1 was abrogated by deletion of the extracellular C-terminal residues 142–152 or by substitution of residues 147–151 with alanines (D_147_VAPR>A_147_AAAA). By contrast, deletion of extracellular N-terminal residues 2–73 did not prevent the binding of clone D1. These data indicate that clone D1 recognizes a C-terminal epitope in mouse NINJ1 (Extended Data Fig. 1d). As a control, we used clone 25 anti-mouse NINJ1 antibody, which recognizes N-terminal residues 22–31 (ref. ^1^). As expected, clone 25 immunolabelling of NINJ1-expressing cells was abrogated by deletion of residues 2–73 of NINJ1 (Fig. 1d).

**Fig. 2:**
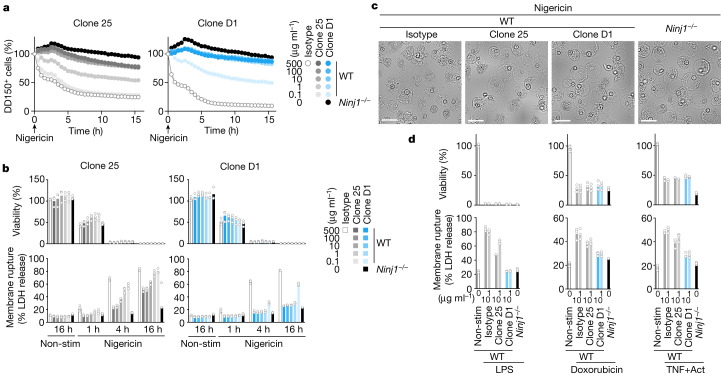
Clone D1 potently inhibits NINJ1-dependent PMR. **a**, The release of DD-150 from Pam3CSK4-primed BMDMs after nigericin stimulation. Data are mean (circles) ± s.d. (shaded area) of biological replicates (*n* = 3 mice); data were generated with bone marrow collected from three mice. **b**,**d**, Viability (top) and LDH release (bottom) in BMDM cultures following pyroptosis induction with nigericin (**b**) or cytoplasmic LPS for 3 h, apoptosis induction with doxorubicin for 6 h or TNF + actinomycin D (Act) for 6 h (**d**). Pyroptotic stimuli were applied to Pam3CSK4-primed BMDMs. **b**,**d**, Bars are the mean of 3 biological replicates (*n* = 3 mice) as in **a**. **c**, Bright-field images of Pam3CSK4-primed BMDMs stimulated with nigericin for 8 h. Scale bar, 25 µm. In **a**–**d**, results are representative of three independent experiments.

To extend our analyses in BMDMs, we monitored NINJ1-dependent PMR by time-lapse live-cell imaging. We loaded Pam3CSK4-primed BMDMs with fluorescein isothiocyanate (FITC)-conjugated 150 kDa dextran (DD-150) and used dye release after nigericin stimulation as an indicator of PMR (Fig. 2a). Clone D1 reduced PMR in BMDMs in a dose-dependent manner compared with an isotype control antibody. The clone D1 Fab also inhibited the release of DD-150 from nigericin-treated BMDMs (Extended Data Fig. 1e). Clone 25 exhibited PMR-blocking activity^1^, but was not as potent as clone D1 (Fig. 2a). Clones D1 and 25 also exhibited dose-dependent inhibition of NINJ1-dependent PMR when measured by LDH release (Fig. 2b, bottom row). As expected, neither clone prevented nigericin-induced cell death, as indicated by the measurement of cellular ATP levels (Fig. 2b, top row). Morphologically, wild-type BMDMs undergoing pyroptosis develop bubble-like herniations that burst in a NINJ1-dependent manner to yield shrunken cellular corpses^1^ (Fig. 2c). However, wild-type BMDMs treated with nigericin in the presence of clone D1 or 25 resembled *Ninj1*^−/−^ BMDMs in that they exhibited a persistent ‘bubble’ morphology (Fig. 2c). Thus, NINJ1-blocking antibodies prevent PMR, but not the formation of membrane herniation during pyroptosis.

**Extended Data Fig. 2:**
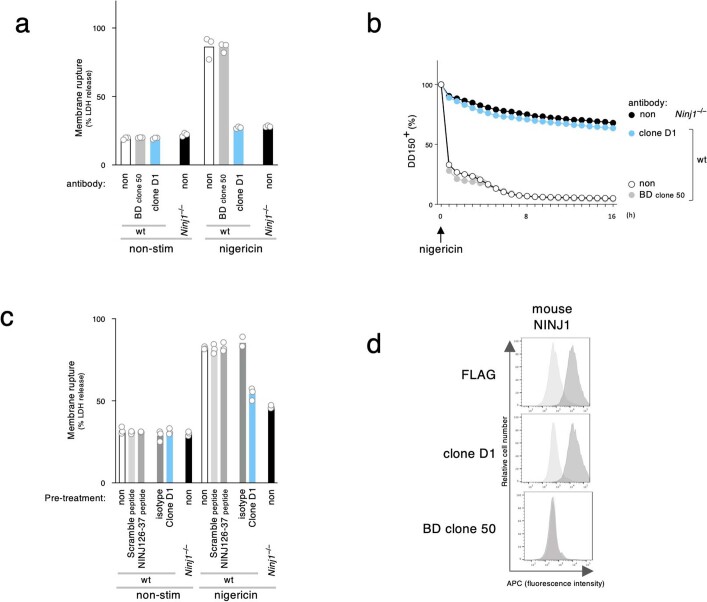
Commercially available anti-NINJ1 antibody. **a**,**c**, Graphs indicate LDH release in cultures of wild-type or *Ninj1*^–/–^ BMDMs after priming, and then stimulation with nigericin for 4 h in the presence or absence (non) of 10 μg/mL indicated antibodies or 200 nM of peptides. Non-stim, non-stimulated control. Bars indicate the mean. BD clone 50, BD Bioscience anti-Ninjurin clone 50. Circles indicate biological replicates (*n* = 3 animals); data were generated with bone marrow from 3 different mice. Results representative of 3 independent experiments. **b**, Graph shows the release of DD-150 from Pam3CSK4-primed wild-type or *Ninj1*^–/–^ BMDMs after nigericin stimulation in the presence or absence (non) of 10 μg/mL indicated antibodies. Data are means (circles) ± s.d. (shaded area) of biological replicates (*n* = 3 animals). Results representative of 3 independent experiments. **d**, Flow cytometry histograms of propidium iodide-negative HEK293T cells surface-stained with indicated antibodies. Cells are mock transfected (light gray) or transfected with mouse NINJ1 (dark gray). Results representative of 3 independent experiments.

PMR is not limited to pyroptosis. BMDMs also undergo GSDMD-independent, but NINJ1-dependent PMR after apoptotic blebbing and shrinkage; this is probably attributable to ATP depletion^1^. Accordingly, *Ninj1*^−/−^ BMDMs released less LDH than wild-type BMDMs following pyroptosis induction with intracellular lipopolysaccharide (LPS) or flagellin, or after apoptosis induction with doxorubicin, venetoclax or TNF plus actinomycin (Fig. 2d and Extended Data Fig. 1f). Clone D1 also attenuated PMR, but not cell death, when wild-type BMDMs were exposed to these pyroptotic or apoptotic stimuli. As expected^1^, neither *Ninj1* deficiency nor clone D1 reduced LDH release following necroptosis induction with TNF plus the pan-caspase inhibitor zVAD (Extended Data Fig. 1f). These results establish anti-mouse NINJ1 antibody clone D1 as a potent inhibitor of PMR associated with apoptosis or pyroptosis. A commercial anti-NINJ1 antibody (BD Bioscience clone 50) and a NINJ1_26–37_ peptide have been used to block mouse NINJ1 in vivo^9–11^, but neither reagent blocked nigericin-induced PMR in BMDMs (Extended Data Fig. 2a–c). Furthermore, BD Bioscience clone 50 did not bind to mouse NINJ1 expressed in HEK293T cells (Extended Data Fig. 2d). Therefore, clone D1 appears to be unique in its ability to potently block NINJ1-dependent PMR (Fig. 1b).

## Clone D1 blocks NINJ1 oligomerization

**Fig. 3:**
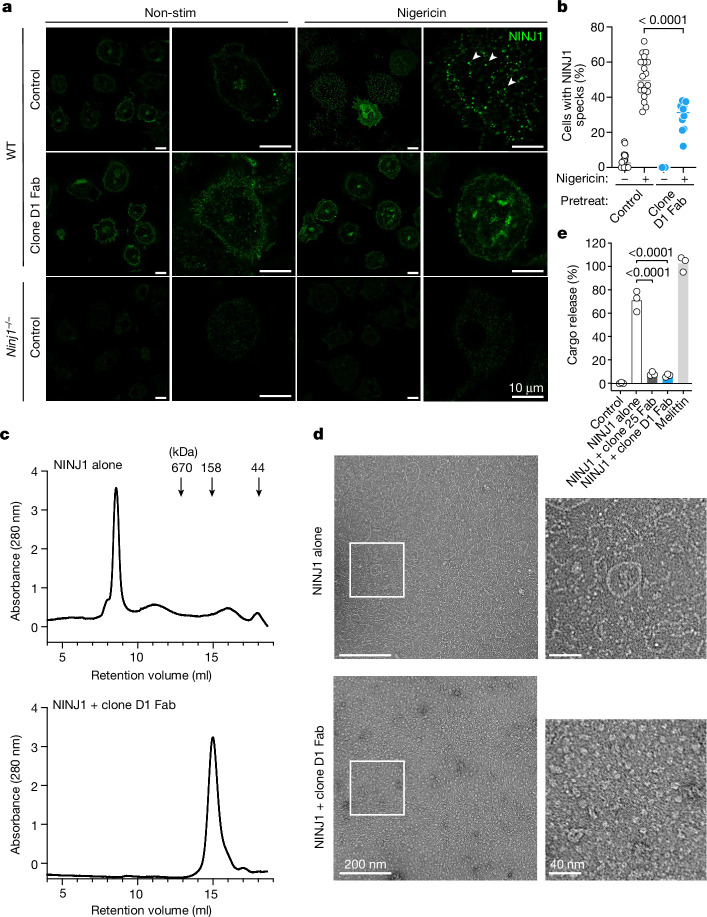
Clone D1 attenuates NINJ1 oligomerization. **a**, Immunolabelling of endogenous NINJ1 in BMDMs after priming with Pam3CSK4 and then stimulation with nigericin for 45 min in the presence or absence (control) of clone D1 Fab. Arrowheads highlight representative NINJ1 specks. **b**, Quantification of the percentage of cells bearing NINJ1 specks in **a**. The small horizontal lines indicate the mean. D1 Fab: *n* = 10 independent samples; other groups: *n* = 20 independent samples. Two-tailed *t*-test, *P* = 0.0000091. **c**, SEC traces for purified NINJ1 or the NINJ1–clone D1 Fab complex. Molecular weight standard marker positions are indicated by arrows. Results representative of three independent experiments. **d**, Negative-stain electron microscopy of NINJ1 or the NINJ1–clone D1 Fab complex in **c**. **e**, Liposome cargo release by the NINJ1 or NINJ1–D1 Fab complex in **c** or NINJ1–clone 25 Fab complex in Extended Data Fig. 3d. Bars show the mean of three independent replicates (circles). Two-tailed Mann–Whitney *U*-test, *P* = 0.0000411 (NINJ1 + clone 25 Fab versus NINJ1 alone), *P* = 0.0000411 (NINJ1 + clone D1 Fab versus NINJ1 alone). 100% cargo release is the total cargo release following addition of 1% cholamidopropyl(dimethylammonio)-2-hydroxy-1-propanesulfonate (CHAPSO).

**Extended Data Fig. 3:**
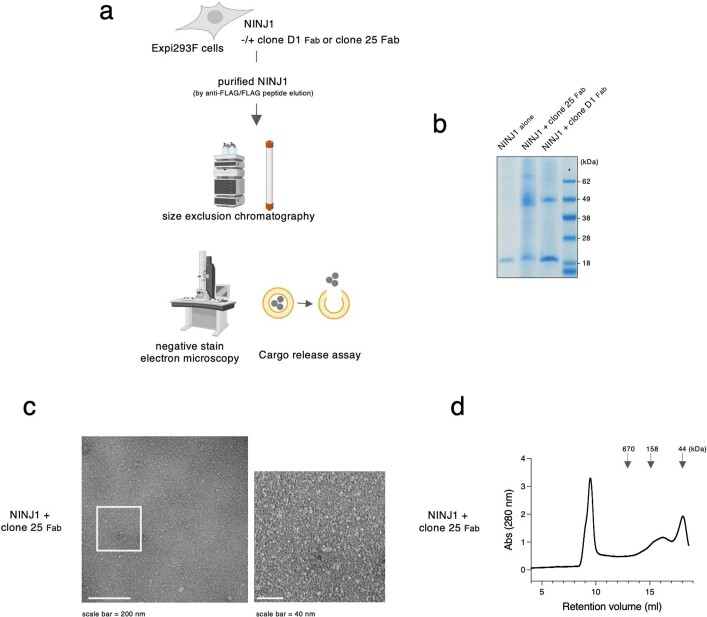
Biochemical analysis for NINJ1-anti-NINJ1 Fab complex. **a**, Schematic of the procedures in Fig. 3c,d,e, and Extended Data Fig. 3b, c, d. **b**, Coomassie blue staining of NINJ1 alone (retention volume 8.5-9 ml) and NINJ1-clone D1 Fab complex (retention volume 15-15.5 ml) of Fig. 3c or NINJ1-clone 25 Fab complex of (**d**) (retention volume 15.5-16 ml). Results representative of 2 independent experiments. **c**, Negative stain electron microscopy of NINJ1-clone 25 Fab complex. Results representative of 2 independent experiments. **d**, Size exclusion chromatography traces for purified NINJ1-clone 25 Fab complex. Results representative of 3 independent experiments. Molecular weight standard marker positions are indicated by arrows.

We hypothesized that clone D1 blocked PMR in BMDMs by preventing the oligomerization of NINJ1^1^. In keeping with such a mechanism, nigericin induced speck-like assemblies of NINJ1 in wild-type BMDMs, but these were less prevalent in the presence of a clone D1 Fab (Fig. 3a,b). We also examined the effect of clone D1 on N-terminally Flag-tagged mouse NINJ1 purified from transiently transfected human Expi293F cells. By size-exclusion chromatography (SEC), purified Flag–NINJ1 migrated as a high molecular weight species (peak at 8.5 ml retention volume) (Fig. 3c and Extended Data Fig. 3a,b). Negative-stain electron microscopy of Flag–NINJ1 revealed that NINJ1 formed oligomeric structures with heterogeneous shapes, including rings, filaments, clusters and arcs up to 200 nm in size (Fig. 3d). By contrast, when Flag–NINJ1 was co-expressed with clone D1 Fab, the purified NINJ1–Fab complex migrated as a lower molecular weight species (peak at 15 ml retention volume) (Fig. 3c and Extended Data Fig. 3a,b) and showed no high-order oligomeric structure formation in negative-stain electron microscopy (Fig. 3d). Thus, binding of clone D1 Fab to NINJ1 prevents it from assembling into larger oligomeric structures.

Purified Flag–NINJ1 added to synthetic liposome membranes caused the release of an encapsulated cargo, whereas the NINJ1–D1 Fab complex did not (Fig. 3e). These data suggest that NINJ1 forms lytic higher-order oligomers, which can be prevented by clone D1. The formation of higher-order oligomeric filaments is not without precedent and has been reported for multiple apoptotic, pyroptotic and necroptotic molecules, including ASC and caspase-8^12,13^. We propose that oligomeric assemblies of NINJ1 mediate PMR in cells and clone D1 binding to NINJ1 prevents these oligomers from forming. Clone 25 resembled clone D1 in preventing NINJ1-dependent cargo release from liposomes (Fig. 3e) and preventing NINJ1 filament formation, as shown by negative-stain electron microscopy (Extended Data Fig. 3c), but was not as efficient as clone D1 at preventing the oligomeric assembly of NINJ1 in SEC analysis (Extended Data Fig. 3d). These data are consistent with clone 25 being a weaker antagonist than clone D1 in cellular PMR assays (Fig. 2a,b). Neither the C-terminal residues of NINJ1 recognized by clone D1 nor the N-terminal residues bound by clone 25 is resolved in the cryo-electron microscopy structure of NINJ1 filaments^14^, suggesting that these regions of NINJ1 are flexible and potentially dispensable for oligomerization. Indeed, alanine mutations within these regions did not suppress membrane damage caused by ectopic expression of NINJ1^1^. Therefore, binding of clone D1 or clone 25 to NINJ1 may prevent NINJ1 oligomerization through steric hindrance.

## Targeting PMR in mouse hepatitis models

**Fig. 4:**
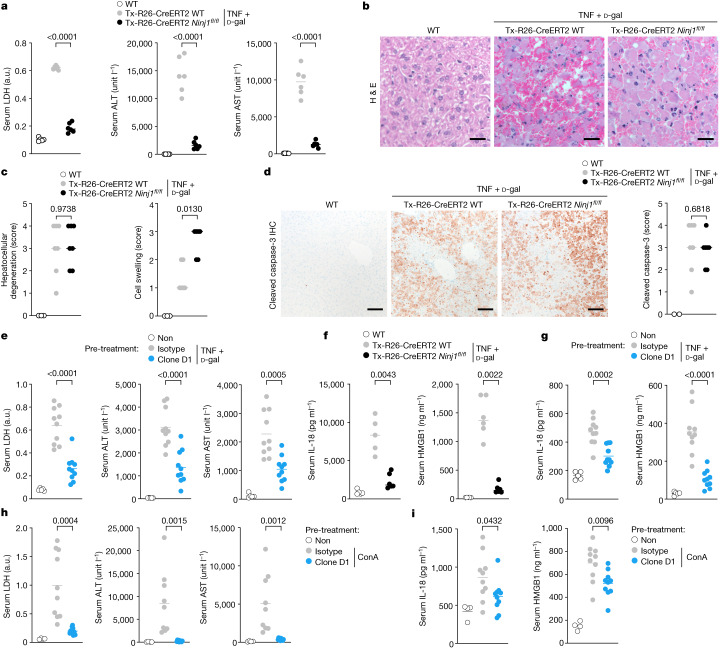
Clone D1 limits NINJ1-dependent PMR and DAMP release in vivo. **a**, Mouse serum LDH, ALT and AST. Where indicated, mice were treated with TNF and d-Gal for 7 h. Untreated wild-type, *n* = 5 mice; tamoxifen-treated groups, *n* = 6 mice. *P* value two-tailed unpaired *t*-test, *P* = 0.0000000000665 (LDH), *P* = 0.00000296 (ALT), *P* = 0.000000000067 (AST). a.u., arbitrary units. **b**, Representative haematoxylin and eosin-stained liver sections of the mice in **a**. Scale bars, 25 µm. **c**, Histological scoring of mouse livers. Untreated wild type: *n* = 3 mice; tamoxifen-treated groups: *n* = 7 (left) or 6 (right) mice. Two-tailed Mann–Whitney *U*-test. **d**, Left, mouse liver sections with immunolabelling of cleaved caspase-3 (brown). Right, qualitative scoring of cleaved caspase-3 labelling. Untreated wild type: *n* = 2 mice; tamoxifen-treated groups: *n* = 7 mice. Two-tailed Mann–Whitney *U*-test. Scale bars, 100 µm. **e**,**h**, Wild-type mouse serum LDH, ALT and AST. Where indicated, mice were dosed with 50 mg kg^−1^ antibody for 2 h before dosing with TNF plus d-Gal for 6 h (**e**) or ConA for 8 h (**h**). Untreated wild type: *n* = 5 (**e**) or *n* = 4 mice (**h**); wild type dosed with antibodies: *n* = 10 mice. Two-tailed unpaired *t*-test. **e,** *P* = 0.00000915 (LDH), *P* = 0.000094 (ALT). **f**, Serum IL-18 and HMGB1 of mice in **a**. Untreated wild type: *n* = 5 mice; tamoxifen-treated *Ninj1*^*+/+*^ *Rosa26*-*creER*^*T2*^: *n* = 5 (left), *n* = 6 (right) mice; tamoxifen-treated *Ninj1*^*fl/fl*^ *Rosa26*-*creER*^*T2*^: *n* = 6 mice. **g**, Serum IL-18 and HMGB1 of mice in **e**. **i**, Serum IL-18 and HMGB1 of mice in **h**. Untreated wild-type: *n* = 5 (**g**) or *n* = 4 (**i**) mice; treated groups: *n* = 10 mice (**g**,**i**). Two-tailed Mann–Whitney *U*-test (**f**), two-tailed unpaired *t*-test (**g**,**i**); *P* = 0.00000682 (**g**). In **a**,**e**–**i**, lines represent the mean and circles represent individual mice; in **c**,**d**, lines represent the median and circles represent individual mice. Source data

**Extended Data Fig. 4:**
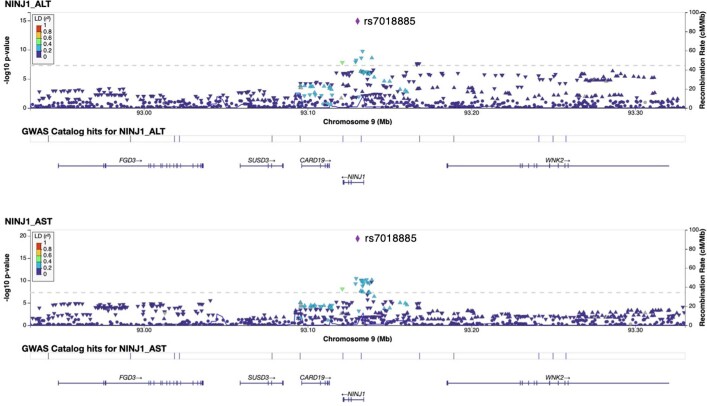
A link between *NINJ1* and lower levels of serum AST and ALT in Genome-wide association studies (GWAS). Regional plot of GWAS (UK Biobank random participants, *n* = 363, 228)^15,42^ showing correlation of a *NINJ1* single nucleotide polymorphism (rs7018885, purple diamond in chromosome 9 locus) with lower serum AST (bottom) or ALT (top). *P* values were obtained from a two-sided test for the null hypothesis that the effect of genotype (at each single nucleotide polymorphism) on the ALT or AST values in a linear regression model was zero. *P* values are not adjusted for multiple tests across the genome.

**Extended Data Fig. 5:**
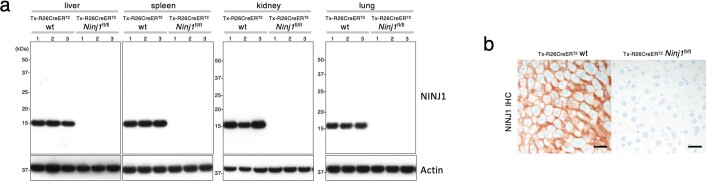
Characterization of tamoxifen-treated *Ninj1*^*fl/fl*^*Rosa26*-CreER^T2^ mice. **a**, Immunoblots of mouse tissues at 2 weeks after tamoxifen dosing. Lanes represent different mice. *n* = 3 mice per genotype. **b**, Immunolabeling of NINJ1 (brown) in mouse liver sections. Scale bar, 25 µm. Results representative of 3 mice per genotype.

**Extended Data Fig. 6:**
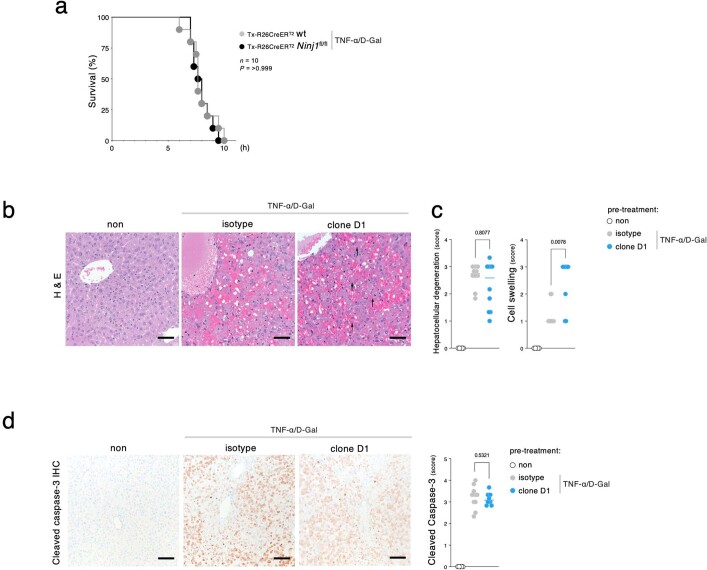
Impact of clone D1 pretreatment on hepatocellular degeneration and caspase-3 cleavage in mice treated with TNF plus D-Gal. **a**, Kaplan–Meier survival plots of mice injected with 25 μg kg^−1^ TNF plus D-Gal. *n* = 10 mice per group. *P* value two-tailed log-rank test. Results representative of 3 independent experiments. **b**, Representative haematoxylin and eosin-stained liver sections of the mice in Fig. 4e. Scale bar, 50 µm. Arrows indicate swollen cells. **c**, Histological scores of hepatocellular degeneration (left) and liver cell swelling (right) for the mice in Fig. 4e. Untreated wild-type, *n* = 5 mice; wild-type dosed with antibody, TNF plus D-Gal, *n* = 10 (left), n = 9 (right) mice. Lines represent the median, circles individual mice. *P* value two-tailed Mann-Whitney *U*-test. **d**, Liver sections from the mice in Fig. 4e after immunolabeling of cleaved caspase-3 (brown). Graph indicates qualitative scoring of cleaved caspase-3 labeling. Untreated wild-type, *n* = 5 mice; wild-type dosed with isotype control antibody, TNF plus D-Gal, *n* = 10 mice; Wild-type dosed with clone D1 and TNF plus D-Gal, *n* = 10 mice. Lines represent the median, circles individual mice. *P* value two-tailed Mann-Whitney *U*-test. Scale bar, 100 µm. Source data

The role of NINJ1-dependent PMR in human disease and inflammation is unclear, but genome-wide association studies^15^ suggest a link between *NINJ1* and reduced serum levels of the liver enzymes alanine aminotransaminase (ALT) and aspartate aminotransferase (AST), two clinically important biomarkers of hepatocellular injury or membrane damage^16,17^ (Extended Data Fig. 4). TNF-induced hepatocyte apoptosis causes liver inflammation, and has been implicated in multiple diseases including hepatocellular carcinoma, ischaemia and viral hepatitis^18^. To address the role of NINJ1 in apoptosis-associated PMR in vivo, we assessed *Ninj1*^*fl/fl*^ *Rosa26*-*creER*^*T2*^ mice in a model of fulminant hepatitis. This mouse strain enables tamoxifen-induced systemic *Ninj1* deletion in adults (Extended Data Fig. 5a,b), and therefore avoids the developmental hydrocephalus that is observed in a significant fraction of *Ninj1*^−/−^ newborns^5^. After tamoxifen treatment, *Ninj1*^*fl/fl*^ *Rosa26*-*creER*^*T2*^ mice and *Rosa26*-*creER*^*T2*^ controls were dosed with TNF and the transcriptional inhibitor d-galactosamine (d-Gal) to induce hepatocyte apoptosis^19–21^. TNF plus d-Gal caused fulminant hepatocellular PMR in *Rosa26*-*creER*^*T2*^ mice as measured by increased serum ALT, AST and LDH (Fig. 4a). The sera of *Ninj1*-deficient mice treated with TNF plus d-Gal contained significantly less ALT, AST, and LDH. Histological analysis of control livers revealed that TNF plus d-Gal caused massive lesions characterized by pyknotic hepatocellular death with haemorrhage (Fig. 4b,c). Immunolabelling showed that these lesions were positive for cleaved caspase-3, a marker of apoptosis (Fig. 4d). *Ninj1* deficiency did not abate hepatocellular degeneration and caspase-3 cleavage induced by TNF plus d-Gal (Fig. 4b–d), consistent with the post-apoptotic role of NINJ1^1^. Although mortality was not delayed in this acute liver injury model (Extended Data Fig. 6a), a greater proportion of *Ninj1*-deficient hepatocytes exhibited the swollen morphology associated with PMR malfunction (Fig. 4b,c). These data indicate that NINJ1 mediates apoptosis-related PMR in vivo.

Next, we determined whether clone D1 could limit liver injury induced by TNF plus d-Gal. Wild-type mice dosed with an isotype control antibody 2 h prior to injection of TNF plus d-Gal exhibited markedly increased serum levels of ALT, AST and LDH, indicative of robust hepatocellular PMR (Fig. 4e). Similar to *Ninj1* deficiency, pre-treatment with clone D1 significantly attenuated TNF plus d-Gal-induced increases in serum LDH, ALT and AST (Fig. 4e). Clone D1 also caused TNF and d-Gal-treated hepatocytes to have a ballooned morphology, whereas degeneration of the liver and the extent of caspase-3 cleavage was similar to that in livers pre-treated with the isotype control antibody (Extended Data Fig. 6b–d). The presence of similar numbers of cleaved caspase-3-positive cells in control and D1-treated (or *Ninj1*-deficient) mice suggests that NINJ1 inhibition does not alter the clearance of dead cells by phagocytes. Indeed, adult *Ninj1*-deficient mice do not develop the spontaneous inflammation that is typical of mice with defective efferocytosis^22^.

TNF-dependent activation of apoptosis in d-Gal-sensitized hepatocytes relies on caspase-8^23^, which also cleaves and activates leaderless interleukin 18 (IL-18)^24–26^, but the mechanism by which IL-18, and potentially other DAMPs, are released from apoptotic cells is not well defined. We found that TNF plus d-Gal increased levels of the DAMPs IL-18 and HMGB1^27^ in the serum in a NINJ1-dependent manner (Fig. 4f,g). *Ninj1* deficiency or pre-treatment of mice with clone D1 prevented the increase in serum IL-18 and HMGB1 after treatment with TNF plus d-Gal. These data strongly suggest that PMR mediated by NINJ1 releases IL-18 into the serum in this model of apoptosis-driven liver injury. This mechanism differs from the one in pyroptotic BMDMs, in which GSDMD pores^28^ suffice to release IL-18 and other small DAMPs independently of NINJ1^1^. Although the role of GSDMD pores in IL-18 release in vivo remains uncertain, our data indicate that both NINJ1-dependent and NINJ1-independent mechanisms may release small DAMPs in disease.

**Extended Data Fig. 7:**
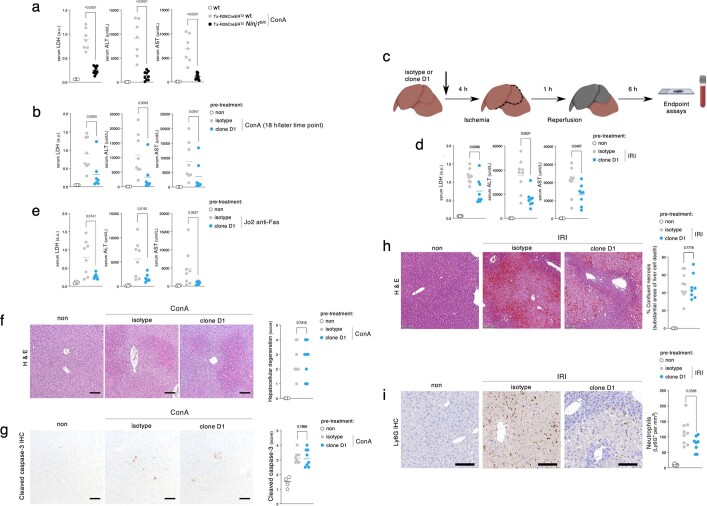
Clone D1 limits NINJ1-dependent PMR and inflammation in hepatitis models. **a**, Mouse serum LDH, ALT, and AST. Where indicated, mice were dosed for 8 h with ConA. Untreated wild-type, *n* = 4 mice; tamoxifen-treated *Ninj1*^+/+^ *Rosa26*-CreER^T2^, *n* = 7 mice; tamoxifen-treated *Ninj1*^fl/fl^ *Rosa26*-CreER^T2^, *n* = 9 mice. a.u., arbitrary units. Lines indicate the mean, circles individual mice. *P* value two-tailed unpaired *t*-test, *P* = 0.00000112 (LDH), *P* = 0.0000427 (ALT), *P* = 0.0000319 (AST). **b**,**e**, Wild-type mouse serum LDH, ALT, and AST. Where indicated, mice were dosed with 50 mg kg^−1^ antibody for 2 h before dosing with 15 mg kg^−1^ ConA for 18 h (**b**) or Jo2 anti-Fas for 5 h (**e**). Untreated wild-type, *n* = 5 mice; wild-type dosed with isotype control antibody, *n* = 8 mice; wild-type dosed with clone D1, *n* = 7 mice. Lines indicate the mean, circles individual mice. *P* value two-tailed Mann-Whitney *U*-test (**b**) and two-tailed unpaired *t*-test (**e**). **c**, Schematic of the IRI procedure in (**d**,**h**,**i**). **d**, Wild-type mouse serum LDH, ALT, and AST. Where indicated, mice were dosed with 50 mg kg^−1^ antibody for 4 h before IRI for 6 h. Untreated sham, *n* = 6 mice; wild-type dosed with isotype control antibody, *n* = 9 mice; wild-type dosed with clone D1, *n* = 8 mice. Lines indicate the mean, circles individual mice. *P* value two-tailed unpaired *t*-test. **f**,**g**, Liver sections with haematoxylin and eosin-staining (**f**) and immunolabeling of cleaved caspase-3 (brown) (**g**) of mice dosed with 50 mg kg^−1^ antibody for 2 h before dosing ConA for 6 h. Scale bar, 100 µm. Graphs indicate qualitative scoring of hepatocellular degeneration (**f**) and cleaved caspase-3 labeling (**g**). Untreated sham, *n* = 4 mice; wild-type dosed with isotype control antibody, *n* = 8 mice; wild-type dosed with clone D1, *n* = 8 mice. Lines indicate the median, circles individual mice. *P* value two-tailed Mann-Whitney *U*-test. **h**,**i**, Liver sections with representative haematoxylin and eosin (**h**) and Ly6G (**i**) staining of the mice in (**d**). Scale bar, 100 µm. Graphs indicate histological scoring of % confluent necrosis (**h**) and Ly6G-positive cells (neutrophils) (**i**). Untreated sham, *n* = 6 mice; wild-type dosed with isotype control antibody, *n* = 9 mice; wild-type dosed with clone D1, *n* = 8 mice. Lines indicate the median, circles individual mice. *P* value two-tailed unpaired *t*-test. Source data

We also investigated the effect of clone D1 in mouse hepatitis instigated by the T cell mitogen concanavalin A^29^ (ConA), Jo2 anti-Fas agonistic antibody^30^ or ischaemia–reperfusion injury^31^. *Ninj1*-deficient mice or mice pre-treated with clone D1 exhibited less serum LDH, ALT, AST, IL-18 and HMGB1 than control mice at 8 or 18 h after ConA dosing (Fig. 4h,i and Extended Data Fig. 7a,b), after hepatic ischaemia–reperfusion injury (Extended Data Fig. 7c,d) and after Jo2 injection (Extended Data Fig. 7e). As expected, clone D1 did not prevent hepatocellular degeneration and the appearance of cleaved caspase-3-positive cells after ConA treatment (Extended Data Fig. 7f,g) or confluent necrosis after ischaemia–reperfusion injury (Extended Data Fig. 7h). Of note, we observed NINJ1-dependent neutrophil recruitment into the damaged liver after ischaemia–reperfusion injury (Extended Data Fig. 7i), in agreement with a recent study^32^ using *Ninj1*-deficient mice. Collectively, these data suggest that NINJ1 mediates hepatocellular PMR and promotes inflammation in vivo.

The identification of antagonist anti-NINJ1 monoclonal antibodies that can prevent the assembly of lytic NINJ1 oligomers and thereby limit PMR and the release of pro-inflammatory DAMPs in vivo indicates that it is possible to target a post-cell death event. Although treatment with clone D1 attenuated PMR in vivo, its efficacy was evaluated in acute mouse hepatitis models. The effect of NINJ1 blockade in settings of chronic inflammation, where protracted DAMP release is expected to exacerbate pathology, will be an exciting area of future investigation, but will require reagents with improved pharmacokinetic properties. We were unable to sustain serum levels of clone D1 long term by repeat dosing.

## Methods

### Mice

All animal procedures were conducted under protocols approved by the Genentech Institutional Animal Care and Use Committee in an Association for Assessment and Accreditation of Laboratory Animal Care (AAALAC)-accredited facility in accordance with the Guide for the Care and Use of Laboratory Animals and applicable laws and regulations. All animal procedures related to hepatic ischaemia–reperfusion injury were conducted under protocols approved by the Animal Care Committee at The Hospital for Sick Children and in accordance with animal care regulation and policies of the Canadian Council on Animal Care. *Ninj1*^−/−^ mice with a C57BL/6N background were described previously^1^. Wild-type mice (which have two alleles of wild-type *Ninj1, Ninj1*^+/+^) were littermates. *Ninj1*^*fl/fl*^ with exon 3 floxed were generated by Ozgene from C57BL/6 ES cells. *Ninj1*^*fl/+*^ mice were genotyped with PCR primers (5′-TAGTTAGTTCAAGCCAGAG, 5′-GCGGTCAGCAGAATAGA, and 5′-CCAAGGAAGCAGGTAC) yielding 396-bp wild-type, 448-bp floxed and 359-bp knockout DNA fragments. *Ninj1*^*fl/fl*^ mice were bred with *Rosa26*^*c*^^*reERT2*/+^ C57BL/6 mice^33^.

For in vivo studies (described below), *Ninj1*^*fl/fl*^ mice were used rather than *Ninj1*^−/−^ mice to avoid the developmental hydrocephalus that is observed in a significant fraction of *Ninj1*^−/−^ newborns^5^. *Ninj1*^*fl/fl*^ *Rosa26*^*creERT2*/+^ and *Ninj1*^+/+^ *Rosa26*^*creERT2*/+^ siblings aged 6–9 weeks were dosed daily by intraperitoneal injection of 80 mg kg^−1^ body weight of tamoxifen in sunflower oil (Millipore Sigma) for 5 consecutive days. Experiments and analyses were performed two weeks after the final dose of tamoxifen.

Mice were housed in individually ventilated cages within animal rooms maintained on a 14 h:10 h, light:dark cycle with ad libitum access to food and water. Animal rooms were temperature and humidity controlled between 20–26 °C and 30–70% humidity, with 10 to 15 room air exchanges per hour.

### Generation of extracellular vesicles and recombinant mouse NINJ1

Expi293F cells (Thermo Fisher Scientific) were co-transfected with pRK-Flag-mNINJ1 (Genentech) and a mammalian expression vector encoding HIV-1–murine leukaemia virus chimeric Gag^34^. Extracellular vesicles were purified from the supernatant 7 days post-transfection using ultracentrifugation^35^.

For recombinant NINJ1 production, Expi293F suspension cells were cultured in Expi293 Expression Medium (Thermo Fisher Scientific) and transfected with pRK-Flag-mNINJ1 using the ExpiFectamine 293 transfection kit (Thermo Fisher Scientific). Frozen cell pellets (50 g) were thawed and washed with a hypotonic buffer containing 20 mM HEPES pH 7.5, 1 mM EDTA, supplemented with leupeptin, benzamidine and protease inhibitor tablets (Roche). Cells were solubilized with 50 mM HEPES pH 7.5, 300 mM NaCl, 1% (w/v) lauryl maltose neopentyl glycol (LMNG, Anatrace), 0.1% (w/v) cholesteryl hemisuccinate (CHS, Anatrace), supplemented with leupeptin, benzamidine and protease inhibitor tablets (Roche) for 1.5 h at 4 °C under gentle agitation. After ultracentrifugation at 185,000*g* for 1 h, the supernatant was gently rotated with anti-Flag M2 affinity resin (Sigma) for 1 h at 4 °C. Unbound proteins were washed away with 10 column volumes of wash buffer A (50 mM HEPES pH 7.5, 300 mM NaCl, 0.1% (w/v) LMNG, 0.01% (w/v) CHS), followed by 10 column volumes of wash buffer B (50 mM HEPES pH 7.5, 150 mM NaCl, 0.005% (w/v) LMNG, 0.0005% (w/v) CHS). Recombinant NINJ1 was eluted with 5 column volumes of wash buffer B supplemented with 300 μg ml^−1^ Flag peptide (Millipore Sigma). Eluate was concentrated in a 50 kDa MWCO concentrator and loaded onto a Superose 6 Increase 10/300 GL column (GE Healthcare) equilibrated with 20 mM HEPES pH 7.5, 150 mM NaCl, 0.005% (w/v) LMNG, 0.0005% (w/v) CHS. Recombinant NINJ1 was conjugated to Alexa Fluor 647 with a Lightning-Link Alexa Fluor 647 kit (Novus Biologicals).

### Anti-mouse NINJ1 antibody generation and screening

*Ninj1*^−/−^ mice were immunized biweekly with mouse NINJ1-expressing extracellular vesicles plus Ribi adjuvant (Millipore Sigma) and mouse plasmids encoding mouse NINJ1 (in pCAGGS vector, Genentech), Flt3L (in pORF vector, Genentech) and mouse GM-CSF (in pORF vector, Genentech). Sera from immunized mice were tested by flow cytometry for reactivity towards mouse NINJ1-expressing BALB/3T3 cells (clone A31, ATCC CCL-163). B cells from the lymph nodes and spleens of immunized mice were enriched using a cocktail of depletion antibodies (biotinylated CD11b at 1:400, CD11c at 1:400, Ly6G/C at 1:400, CD49b at 1:400, TER119 at 1:100, CD4 at 1:200, CD8b.2 at 1:200, CD11b at 1:400 from BD Biosciences) and magnetic Streptavidin beads (Miltenyi Biotec). Enriched cells were stained with PE-Cy7-conjugated rat anti-mouse IgM (BD Pharmingen, 1:50-100), rat anti-mouse B220 eFluor 450 (Thermo Fisher Scientific, 1:50), and AF647-labelled mouse NINJ1. Approximately 15,000 NINJ1-bound IgM^–^B220^+^ B cells were sorted as single cells into 96-well plates containing supplemented RPMI 1640 medium (Thermo Fisher Scientific) and EL-4-B5 feeder cells (Roche). After 8 to 10 days in culture, supernatants were screened for mouse NINJ1-reactive IgGs using an IgG ELISA (Rockland) and flow cytometry against NINJ1-expressing 3T3 cells. B cells producing NINJ1-reactive IgGs were the starting point for the generation of 217 recombinant monoclonal antibodies using published methods^36^. In brief, RNA was extracted from the B cells using a MagMax-96 Total RNA Isolation Kit (Thermo Fisher Scientific). Variable light (V_L_) and variable heavy (V_H_) domains were PCR-amplified from cDNA using a forward barcoded primer set recognizing the leader sequence of most known mouse variable genes, and a barcoded reverse primer recognizing the constant domain^37^. Individual purified V_L_ and V_H_ PCR products were pooled for next-generation sequencing library preparation using the Ovation Library System for Low Complexity Samples kit (Nugen). A MiSeq sequencer (Illumina) was used for 2× 300-bp paired sequencing. V_H_ and V_L_ sequences were synthesized (Genscript) and cloned into pRK mammalian expression vectors encoding the mouse γ2a and k constant domains, respectively^38^. Recombinant antibodies were transiently expressed in CHO cells and purified on a protein A column^38^. In brief, CHO cells (Genentech) were seeded at 0.4–0.8 × 10^6^ cells per ml. Three or four days later, cells were transfected with plasmids by using PEIPro (Polyplus) according to the manufacturer’s instructions. Culture supernatants were harvested 14 days post-transfection. Recombinant antibodies were screened for NINJ1 inhibitory activity by LDH release assay in BMDMs. Antibodies (1 µg ml^−1^) were added to Pam3CSK4-primed BMDMs for 15 min prior to nigericin stimulation. At 16 h post stimulation, supernatants were collected for LDH release assays.

### Reagents

Ultra-pure LPS (*Escherichia coli* O111:B4, InvivoGen), Pam3CSK4 (InvivoGen), nigericin (InvivoGen), ultra-pure flagellin (from *Pseudomonas aeruginosa*, InvivoGen), venetoclax (TOCRIS), doxorubicin (Enzo Life Sciences), mouse TNF (Genentech), actinomycin D (EMD Millipore), z-VAD-FMK (zVAD; Promega). Antibodies used for western blotting were rabbit anti-mouse NINJ1 clone 25 (Ninj1-25, Genentech, 0.2 µg ml^−1^)^1^, β-actin HRP (AC-15, Novus Biologicals, 0.1 µg ml^−1^), HRP–anti-rabbit F(ab′)_2_ fragment (Jackson Immunoresearch, 1:5,000), and HRP–anti-rabbit Fc fragment (Jackson Immunoresearch, 1:5,000). A list of all antibodies used in this manuscript is provided in Supplementary Table 1. cDNAs encoding N-terminally Flag-tagged NINJ1 (full-length, delta 2–73, delta 142–152 and mutant D_147_VAPR → A_147_AAAA) were cloned into pcDNA3.1 Zeo(+) (Thermo Fisher Scientific).

### Cell line authentication and quality control

Cell lines were authenticated by short tandom repeat (STR) profiling and regular single nucleotide polymorphism (SNP) fingerprinting. STR profiles are determined for each line using the Promega PowerPlex 16 System. This is performed once and compared to external STR profiles of cell lines (when available) to determine cell line ancestry. SNP profiles are performed each time new stocks are expanded for cryopreservation. Cell line identity is verified by high-throughput SNP profiling using Fluidigm multiplexed assays. SNPs were selected based on minor allele frequency and presence on commercial genotyping platforms. SNP profiles are compared to SNP calls from available internal and external data (when available) to determine or confirm ancestry. All cells are tested for mycoplasma prior to and after cell cryopreservation using two methods, to avoid false positive or negative results: Lonza Mycoalert and Stratagene Mycosensor. Cell growth rates and morphology were also monitored for any batch-to-batch changes.

### BMDM stimulations

Mouse bone marrow cells were differentiated into macrophages in Dulbecco’s modified Eagle’s medium (DMEM) supplemented with 10% (v/v) low-endotoxin fetal bovine serum (FBS; Omega Scientific) and 20% (v/v) L929-conditioned medium for 5 days. For stimulation, cells were replated overnight at 1.0 × 10^5^ cells per well in 96-well plates. For inflammasome stimulations, cells were primed with Pam3CSK4 (1 μg ml^−1^) for 5 h and then stimulated with 10 μg ml^−1^ nigericin in Opti-MEM I media (Thermo Fisher Scientific). For flagellin or LPS electroporation^39^, 1.0 × 10^6^ Pam3CSK4-primed BMDMs were electroporated with 1.0 μg LPS or 0.2 μg flagellin in 100 μl R buffer using a neon 100-μl tip with 1,720 voltage, 10 width, 2 pulse settings. Electroporated cells were added to 990 μl Opti-MEM I medium and cultured for 2 h. BMDMs treated with venetoclax (25 μM for 6 h), doxorubicin (10 μM for 6 h), TNF plus actinomycin D (20 ng ml^−1^ TNF, 500 ng ml^−1^ actinomycin D for 6 h), or TNF plus zVAD (100 ng ml^−1^ TNF, 20 μM zVAD for 16 h) were not primed. For lysis controls, cells were lysed with 0.25% Triton-X100 in medium. Where indicated, BMDMs were cultured with the indicated concentration of anti-NINJ1 clone 25^1^ (mouse IgG2a; Genentech), anti-NINJ1 clone D1 (mouse IgG2a; Genentech), anti-ninjurin clone 50 (BD Biosciences BD610777, raised against human NINJ1), or an isotype control mouse IgG2a (Thermo Fisher Scientific). Prior to addition to cells, anti-ninjurin clone 50 and isotype control mouse IgG2a antibodies were dialysed against PBS to remove sodium azide using Slide-A-Lyzer MINI Dialysis Device with a 10K MWCO (Thermo Fisher Scientific) according to the manufacturer’s instructions. Synthetic peptides used were mouse NINJ1 amino acids 26–37 (PPRWGLRNRPIN, Genentech) and its sequence-scrambled analogue (PWPPRRNRNGLI, Genentech). BMDMs were pre-treated with antibody or peptide for 10 min prior to addition of treatment.

### PMR and viability assays

Culture medium was analysed for LDH release with the CytoTox 96 Non-Radioactive Cytotoxicity Assay (Promega). CellTiter-Glo reagent (ATP assay, Promega) was used for detection of viable cells. Data for LDH and CellTiter-Glo assays was collected with an EnVision 2105 multimode plate using EnVision Manager 1.14.3049.1193 (PerkinElmer).

### Flow cytometry

293T cells (ATCC, CRL-3216) cells were transfected with NINJ1 expression plasmids using Lipofectamine 2000 (Thermo Fisher Scientific). Cells were stained with the following monoclonal antibodies: anti-NINJ1 clone 25 mIgG2a (Genentech, 10 µg ml^−1^), anti-NINJ1 clone D1 mIgG2a (Genentech, 10 µg ml^−1^), anti-Flag-M2 (Millipore Sigma, 1:100), anti-ninjurin clone 50 (BD Biosciences, 10 µg ml^−1^). Primary staining was followed by APC-conjugated anti-mouse IgG secondary (Thermo Fisher Scientific, 1:300), and then propidium iodide (PI; 2.5 µg ml^−1^; BD Biosciences). Live PI^–^ cells were analysed in a FACSymphony (Becton Dickinson). Data was acquired using BD FACSDiva Software v9.1, and analysed using FlowJo v10.8.1. Representative FACS gating strategies and contour plots with outliers are shown in Supplementary Figs. 2–4.

### DD-150 dye-release assay

BMDMs were loaded with fluorescein isothiocyanate-dextran (DD-150, Millipore Sigma) using a 100 µL Neon tip (Thermo Fisher Scientific). 5.0 × 10^6^ BMDMs were electroporated in 120 µl R buffer plus 12 µl 50 mg ml^−1^ DD-150. Prior to plating, BMDMs were washed with high-glucose DMEM. Following stimulation, BMDMs were imaged in an IncuCyte S3 (Essen BioScience) at 10X magnification.

### Imaging of BMDM morphology

BMDMs were plated on glass-bottom 96 MicroWell Optical Plates (Thermo Fisher Scientific). Pam3CSK4-primed BMDMs were stimulated with 10 μg ml^−1^ nigericin in the presence or absence of 10 µg ml^−1^ anti-NINJ1 antibody. Plates were imaged using a 60× Plan Fluor objective on an ImageXpress Micro Confocal system running the MetaXpress v6.5.4.532 software and equipped with an environmental controller and gas mixer to maintain cells at 37 °C and 5% CO_2_. Images of the bright-field and transmitted light were imaged overnight every 15 min. Representative images at the 8 h time point were processed using the using the scikit-image 0.19.2 python package.

### Immunofluorescence

Clone D1 variable domains were cloned into the human Fab expression vector 1AP39.hIgG1.D.Fab (Genentech). Protein was expressed in *E. coli* and purified with a low endotoxin level (<0.07 EU mg^−1^). Pam3CSK4-primed BMDMs were cultured with 50 μg ml^−1^ D1 Fab and then stimulated with nigericin on glass-bottom 96 MicroWell Optical Plates (Thermo Fisher Scientific). Cells were fixed with 4% paraformaldehyde in PBS and then permeabilized with 0.1% Tween-20. Cells were blocked in PBS supplemented with 0.2% fish gelatin (Millipore Sigma), 3% Bovine Serum Albumin and 0.1% Tween-20 for 1 h at room temperature, and then labelled with anti-mouse NINJ1 clone 80 (rabbit IgG2b raised against the N-terminal extracellular domain; Genentech, 2 µg ml^−1^) at 4 °C overnight. Bound antibody was revealed with an AF488-conjugated anti-rabbit secondary (Thermo Fisher Scientific, 1:200) at room temperature for 1 h. High-resolution images were acquired with a Leica SP8X confocal laser scanning microscope running Leica LAS X v3.5.7 software and equipped with a white light laser and a HC PL APO CS2 oil immersion 40X lens of numerical aperture 1.3.

### Transient expression of NINJ1 in HEK293T cells

cDNAs encoding untagged human or mouse NINJ1 were cloned into pcDNA3.1 Zeo(+). HEK293T cells (2.6 × 10^4^) were transfected with 50 ng plasmid plus 0.16 μl Lipofectamine 2000 per well in 96-well plates in the presence of 20 or 200 μg ml^−1^ of isotype control antibody, anti-NINJ1 clone D1 (Genentech), or anti-NINJ1 clone D1 Fab (Genentech). YOYO-1 dye (Thermo Fisher Scientific) was added at a final concentration of 200 nM at the time of transfection. IncuCyte S3 images were scanned in the green channel every hour for at least 16 h and at 10× magnification. Nuclear-ID Red DNA stain (Enzo Life Sciences) was added after the last time point and scanned in the red channel. IncuCyte S3 2019A software was used to determine the total number of YOYO-1^+^ cells and Nuclear-ID^+^ cells (total cells). The percentage of YOYO-1^+^ cells was calculated as the number of YOYO-1^+^ cells divided by the total number of Nuclear-ID^+^ cells.

### Size-exclusion chromatography

The C-terminally His_6_-tagged heavy chain Fab region (VH-CH1) and the untagged light chain of anti-NINJ1 clone D1 and clone 25 were cloned into the mammalian expression vector pRK5J (Genentech). Expi293F cells were transfected with pRK-Flag-mNINJ1 alone, or co-transfected with pRK-Flag-mNINJ1, pRK5J-His_6_-VH-CH1 clone D1 (or pRK5J-His_6_-VH-CH1 clone 25), and pRK5J-light chain clone D1 (or pRK5J-light chain clone 25) in a 2:1:2 plasmid ratio. NINJ1, the NINJ1-clone D1 Fab complex, or the NINJ1-clone 25 Fab complex was purified with anti-Flag M2 resin (described above). For comparative SEC analysis, similar protein amounts and volume of NINJ1, NINJ1–clone D1 Fab complex, or NINJ1–clone 25 Fab complex were injected onto a Superose 6 Increase 10/300 GL column (GE Healthcare). Data was collected with Unicorn 7.6 (Cytiva).

### Negative-stain electron microscopy

EM grids (400 mesh copper with continuous carbon, Electron Microscopy Sciences) were glow discharged for 15 s at 10 mA using a GloQube glow discharge system (Quorum) before applying 4 μl of sample diluted to approximately 0.1 mg ml^−1^ in SEC buffer (20 mM HEPES pH 7.5, 150 mM NaCl, 0.005% (w/v) LMNG, 0.0005% (w/v) CHS). After 1 min of incubation, the grid was washed with distilled, deionized water, and stained with 1% uranyl acetate. After drying completely, the grids were imaged in a Talos F200C equipped with a 4k × 4k Ceta 16M Camera (Thermo Fisher Scientific) using SerialEM Version 3.9.0. Images were recorded at a nominal magnification of 45,000× (3.2 Å per pixel).

### Liposomal cargo-release assay

Stocks of 1-palmitoyl-2-oleoyl-glycero-3-phosphocholine (POPC, Avanti Polar Lipids) and 1-palmitoyl-2-oleoyl-*sn*-glycero-3-phospho-l-serine (sodium salt) (POPS, Avanti Polar Lipids) were prepared in chloroform from dry powder. A lipid mixture of 80% POPC and 20% POPS was generated, freeze dried, and hydrated with a solution of 10 mM Tris pH 8.0, 150 mM NaCl containing the cargo, LANCE Eu-W1024 Biotin (PerkinElmer). The suspension was sonicated in a water bath, freeze-thawed, and extruded using a Mini Extruder (Avanti Polar Lipids) fitted with a Nucleopore 0.4 μm membrane (Whatman) to yield large unilamellar vesicles. The liposomes were purified by eluting through a column packed with Pierce Streptavidin Agarose resin (Thermo Fisher Scientific). Liposomes were destabilized by addition of 0.0005% LMNG/0.00005% CHS (Anatrace). Cargo-release assay was set up by mixing destabilized liposomes (25 μM in lipid concentration, diluted from 6.4 mM stock) with 8 μM NINJ1 purified according to the protocol above or 0.4 μM Melittin peptide (Anaspec). Streptavidin-Alexa Fluor 647 (Thermo Fisher Scientific) was added to each well at a final concentration of 1 μM. Liposomes mixed with 0.0005% LMNG in ddH_2_O were used as a control. All samples were loaded into wells of a ProxiPlate (PerkinElmer). Time-resolved readout was recorded on an EnVision 2105 multimode plate reader (PerkinElmer) using EnVision Manager 1.14.3049.1193 (PerkinElmer). A pre-read of each plate was taken immediately after loading the wells, and each plate was subsequently incubated overnight at room temperature. After ~15 h of incubation, another reading was taken, followed by full digestion of the liposomes by addition of 1% CHAPSO (Anatrace) to each well. A final read was recorded representing 100% cargo release. Results were converted to percentage cargo released per well and background control subtracted.

### Stimulant-induced liver injury models

In the TNF plus d-Gal model, liver injury was induced in female mice aged 8 to 14 weeks by intraperitoneal injection of 700 mg kg^−1^ d-Gal (Millipore Sigma) and 30 μg kg^−1^ mouse TNF (Genentech) unless otherwise indicated. Serum was collected after 6–7 h. Where indicated, 8 to 14 weeks old age matched C57BL/6J female (Jackson Labs) mice were dosed by intraperitoneal injection of 50 mg kg^−1^ body weight of mouse anti-NINJ1 clone D1 antibody or isotype control anti-gp120 mouse IgG2a monoclonal antibody (Genentech) at 2 h before TNF plus d-Gal. For ConA or anti-Fas mAb (Jo2) treatment, liver injury was induced in males aged 9 to 11 weeks by intravenous injection of either 20 mg kg^−1^ body weight of ConA (Millipore Sigma) or 0.5 μg g^−1^ body weight of anti-Fas (anti-CD95 clone Jo2, BD Biosciences), respectively, unless otherwise indicated. Serum was collected after 8 or 18 h for ConA, and after 5 h for anti-Fas. For antibody pre-treatment, 9 to 11 weeks old C57BL/6N male mice (Charles River Labs) were treated with the indicated monoclonal antibodies as described above. Serum ALT and AST were measured in a serum chemistry analyser (Beckman Coulter AU480). LDH in serum was measured with the CytoTox 96 Non-Radioactive Cytotoxicity Assay. Enzyme-linked immunosorbent assays (ELISAs) were used to assay IL-18 (clone 74 and clone 93-10C, MBL International) and HMGB1 (IBL). LDH assay and IL-18 and HMGB1 ELISA data was collected with an EnVision 2105 multimode plate using EnVision Manager 1.14.3049.1193 (PerkinElmer).

### Histology

Haematoxylin and eosin-stained sections of *Rosa26*^*creERT2*/+^ livers were scored for hepatocellular degeneration on a four-point scale based on the amount of viable tissue present as follows: (1) multifocal hepatocellular injury with preservation of bridging portal tracts, (2) intermixed populations of viable and degenerate cells throughout the liver, (3) bridging hepatocellular injury with only the preservation of peri-portal hepatocytes, or (4) panlobular hepatocellular degeneration with loss of lobular architecture. For scoring hepatocellular degeneration in antibody-treated mice, six lobes were scored according to the following criteria: (0) no significant hepatocellular degeneration, (1) multifocal cell death without loss of architecture, (2) non-bridging lobular hepatocellular degeneration and loss of architecture, (3) bridging hepatocellular degeneration with loss of architecture, or (4) diffuse hepatocellular degeneration. Scores from the six lobes were averaged for a final score. To assess the persistence of swollen, degenerate hepatocytes, livers were scored based on the predominant features of either cell loss with sinusoidal haemorrhage or persistence of swollen degenerate hepatocytes. A three-tier scoring system was applied: (1) predominant hepatocellular loss with sinusoidal haemorrhage, (2) mix of hepatocyte loss and haemorrhage with regional aggregates of swollen, degenerate hepatocytes, or (3) predominant preservation of swollen, degenerate hepatocytes. Scoring was performed in a random, blinded manner.

### Immunohistochemistry

Formalin-fixed, paraffin-embedded sections of liver were immunolabelled with rabbit anti-cleaved caspase-3 antibody (Asp175, Cell Signaling Technologies, 0.05 μg ml^−1^) or rabbit anti-NINJ1 clone 80 (5 μg ml^−1^) using a discovery IHC platform (Roche). Conditions included CC1 standard antigen retrieval (Roche), OmniMap detection (Roche) with diaminobenzidine, and haematoxylin counterstain. Immunohistochemistry and histology images were acquired with Leica Application Suit v4.6.0. Cleaved caspase-3 immunolabelling was scored according to the following matrix: (1) multifocal individual or aggregates of labelled cells, (2) either extensive intermix of labelled and unlabelled cells or centrilobular labelling with variable bridging, (3) extensive bridging cleaved caspase-3 expression with only rims of unlabelled peri-portal hepatocytes, (4) diffuse hepatocellular labelling. Scoring was performed in a random and blinded manner. NINJ1 immunolabeling was qualitatively assessed in an unblinded manner.

### Ischaemia–reperfusion liver injury

Mixed-sex cohorts of 6 to 10 weeks old age C57BL/6J mice (Jackson Labs) were used in a 70% segmental ischaemia–reperfusion model^40^. Under isoflurane anaesthesia, a sagittal midline laparotomy was made, and a clamp placed on the portal vein and the hepatic artery to block blood flow to the left and medial lobes of the liver. The clamp was removed after 1 h to allow for reperfusion and the animal returned to their home cage. Following 6 h of reperfusion, the animal was euthanized by cardiac puncture under general anaesthesia and tissue collected for analysis. Sham laparotomy where the vascular pedicle was exposed but not clamped was used as a negative control. Where indicated, mice were dosed by intraperitoneal injection of 50 mg kg^−1^ antibodies 4 h before induction of ischaemia as described above. Animals were randomized to group and analyses blinded. Serum LDH, ALT, AST levels were measured as described above. For histology, all ischaemic and reperfused liver lobes were collected, paraffin-embedded, sectioned at a thickness of 4 µm prior to staining with haematoxylin and eosin. Neutrophils were with rabbit anti-mouse Ly6G (Cell Signaling Technology, 1:100) followed by biotinylated anti-rabbit secondary antibody (Vector Laboratories, 1:200) and ABC (Vector Laboratories). DAB (Vector Laboratories) was used to detect Ly6G staining. Tissue specimen processing and staining were conducted at the Spatio-Temporal Targeting and Amplification of Radiation Response (STTARR) Innovation Centre. Slides were imaged using a 3DHistech Pannoramic Flash II Slide Scanner and visualized using either 3DHISTECH CaseViewer (RRID: SCR_017654) or HALO Image Analysis Platform (RRID: SCR_018350; indica labs). To evaluate confluent necrosis within the liver samples, the DenseNet classifier supervised machine learning algorithm (HALO Image Analysis Platform) was trained to detect substantial areas of liver cell death using the haematoxylin and eosin stain and applied to the entire sample. Neutrophils (Ly6G-positive) were counted using QuPath (RRID: SCR_018257). Statistical testing was calculated using Prism 9.5.1 (GraphPad Software). Presented data are representative of at least three independent experiments. All collected data was analysed and a *P* value < 0.05 was considered statistically significant.

### Western blots

Tissues were lysed in RIPA buffer (50 mM Tris pH 7.5, 150 mM NaCl, 2 mM EDTA, 1% NP-40, 0.5% SDS, 1× cOmplete Protease Inhibitor (Roche Applied Science) and PhosSTOP phosphatase inhibitor (Millipore Sigma)) at 4 °C for 30 min. Tissues were mechanically disrupted with a bead mill homogenizer (Omni International) and insoluble material was removed by centrifugation at 20,000*g* before addition of NuPAGE LDS sample buffer 4X (Thermo Fisher Scientific). Raw images of uncropped gels are provided in Supplementary Fig. 1.

### Genome-wide association studies

Regional plots were generated using LocusZoom^41^ and genome-wide association study (GWAS) data that used UK Biobank random participant samples (*n* = 363,228) and 35 blood and urine biomarkers^15,42^ (10.1038/s41588-020-00757-z).

### Statistics and figure preparation

Unless otherwise specified, presented data are representative of at least two independent experiments and means are of at least three biological replicates. Statistical analyses and number of samples (*n*) are given in each figure panel. Mann–Whitney *U*-tests, *t*-tests and log-rank tests were performed using GraphPad Prism 9.5.1 (GraphPad Software).

No statistical methods were used to predetermine sample size. Sample sizes were chosen based on prior experience and pilot experiments for detecting statistically significant differences between conditions. For in vivo studies involving tamoxifen-treated animals, groups were determined by genotype rather than treatment, and therefore not randomized. For TNF plus d-Gal, anti-Fas Jo2, and ConA in vivo studies involving wild-type mice, animals were age- and sex- matched and randomized to group. Experimental groups were assessed in the same experiment with control groups to eliminate covariates. For animal procedures related to hepatic ischaemia–reperfusion injury mixed-sex cohorts were used; animals were randomized to group and analyses blinded.

### Reporting summary

Further information on research design is available in the Nature Portfolio Reporting Summary linked to this article.

## Online content

Any methods, additional references, Nature Portfolio reporting summaries, source data, extended data, supplementary information, acknowledgements, peer review information; details of author contributions and competing interests; and statements of data and code availability are available at 10.1038/s41586-023-06191-5.

## Supplementary information

Supplementary Figures This file contains Supplementary Figs. 1-4.

Reporting Summary

Supplementary Table 1 A list of all antibodies used in this study.

## Source data

Source Data Fig. 4

Source Data Extended Data Fig. 6

Source Data Extended Data Fig. 7

## Data Availability

The datasets generated during and/or analysed during the current study are available from the corresponding authors upon reasonable request. GWAS data were obtained from the UK Biobank study^15^. Source data are provided with this paper.
