# Author Correction: Inhibiting membrane rupture with NINJ1 antibodies limits tissue injury

**DOI:** 10.1038/s41586-025-09955-3

**Published:** 2025-11-27

**Authors:** Nobuhiko Kayagaki, Irma B. Stowe, Kamela Alegre, Ishan Deshpande, Shuang Wu, Zhonghua Lin, Opher S. Kornfeld, Bettina L. Lee, Juan Zhang, John Liu, Eric Suto, Wyne P. Lee, Kellen Schneider, WeiYu Lin, Dhaya Seshasayee, Tushar Bhangale, Cecile Chalouni, Matthew C. Johnson, Prajakta Joshi, Jan Mossemann, Sarah Zhao, Danish Ali, Neil M. Goldenberg, Blayne A. Sayed, Benjamin E. Steinberg, Kim Newton, Joshua D. Webster, Ryan L. Kelly, Vishva M. Dixit

**Affiliations:** 1 Department of Physiological Chemistry, Genentech, South San Francisco, CA USA; 2 Department of Structural Biology, Genentech, South San Francisco, CA USA; 3 Department of Antibody Engineering, Genentech, South San Francisco, CA USA; 4 Department of Translational Immunology, Genentech, South San Francisco, CA USA; 5 Department of Human Genetics, Genentech, South San Francisco, CA USA; 6 Department of Pathology, Genentech, South San Francisco, CA USA; 7 Department of Biomolecular Resources, Genentech, South San Francisco, CA USA; 8 Program in Cell Biology, Hospital for Sick Children, Toronto, Ontario Canada; 9 Program in Neuroscience and Mental Health, Hospital for Sick Children, Toronto, Ontario Canada; 10 Department of Anesthesia and Pain Medicine, Hospital for Sick Children, Toronto, Ontario Canada; 11 Division of General Surgery, Hospital for Sick Children, Toronto, Ontario Canada

**Keywords:** Immune cell death, Apoptosis

Correction to: *Nature* 10.1038/s41586-023-06191-5 Published online 17 May 2023

In the version of this article initially published, due to a figure preparation error, in Fig. 3a, bottom row, the fourth image on the right was an inadvertent duplicate of the second image from left in the bottom row. The figure has been amended in the HTML and PDF versions of the article.

