# Supplementary material for: Inhibiting membrane rupture with NINJ1 antibodies limits tissue injury
Source: Nature. 2023 May 17;618(7967):1072–7. doi: 10.1038/s41586-023-06191-5 (PMC10307625; doi:10.1038/s41586-023-06191-5)

nature portfolio

https://doi.org/10.1038/s41586-023-06191-5

Supplementary information

Inhibiting membrane rupture with NINJ1 antibodies limits tissue injury

In the format provided by the
authors and unedited

Supplementary Figure 1. Raw images of uncropped gels

Source Data, Extended Data Fig. 3b

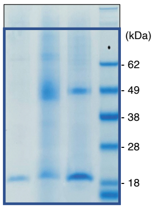

Source Data, Extended Data Fig. 5a

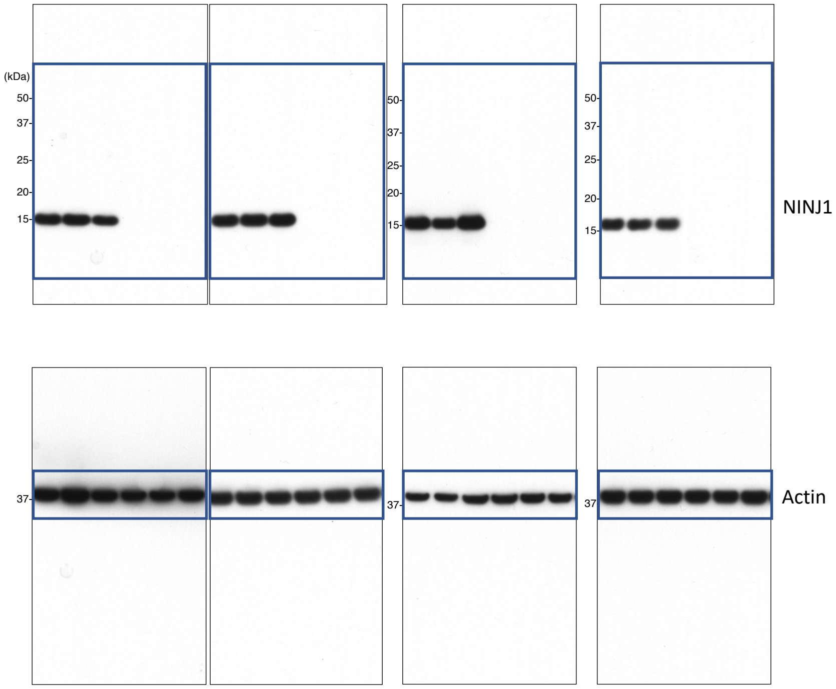

Supplementary Figure 2. Flow cytometry gating strategy

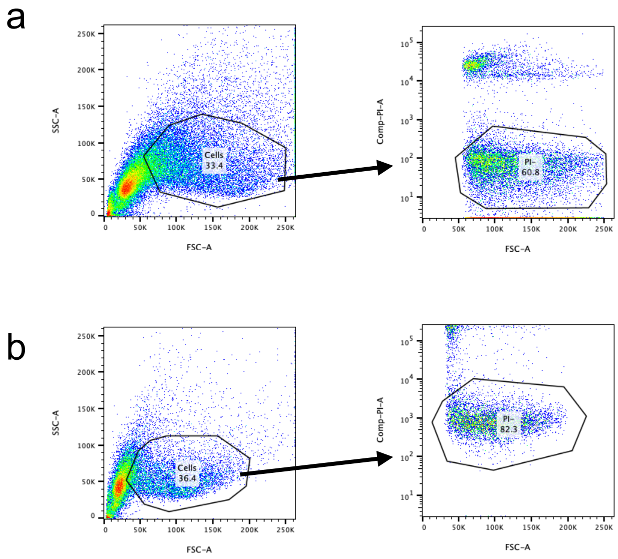

a, Flow cytometry gating strategy to sort PI- cells used for Fig. 1d and Extended Data Fig. 1c.
b, Flow cytometry gating strategy to sort PI- cells used for Extended Data Fig. 2d.

Supplementary Figure 3. Flow cytometry contour plots for Fig. 1d

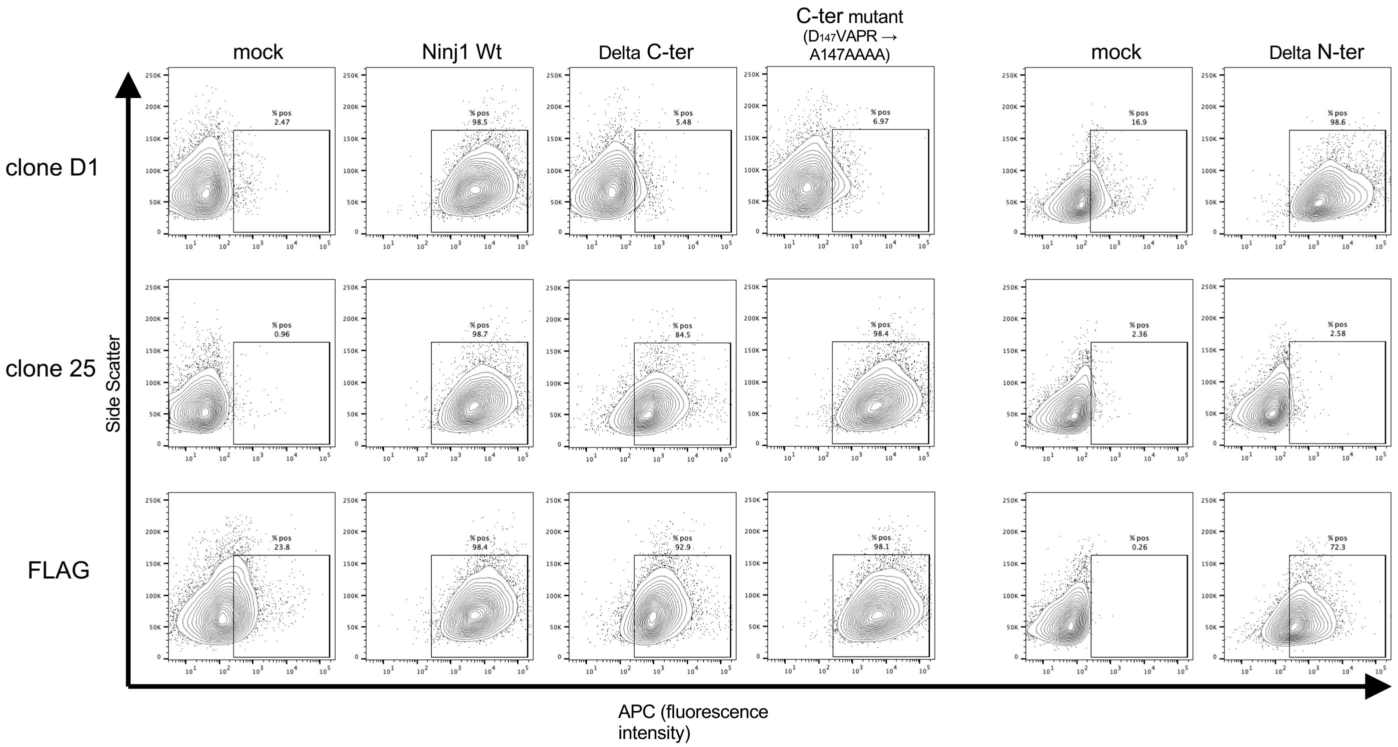

Supplementary Figure 4. Flow cytometry contour plots for Extended Data Figs. 1c and 2d

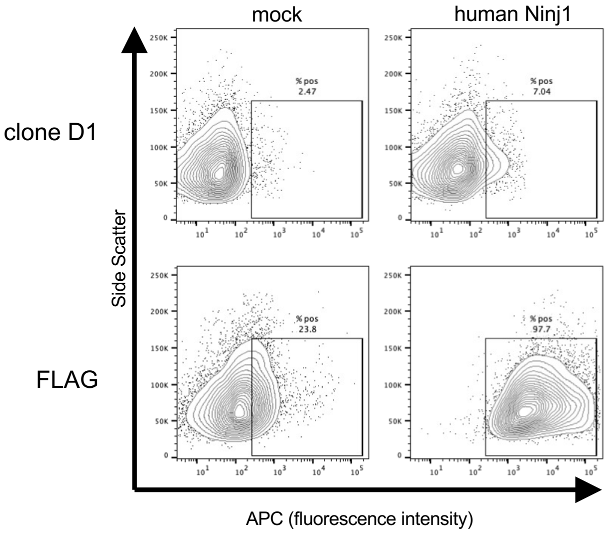

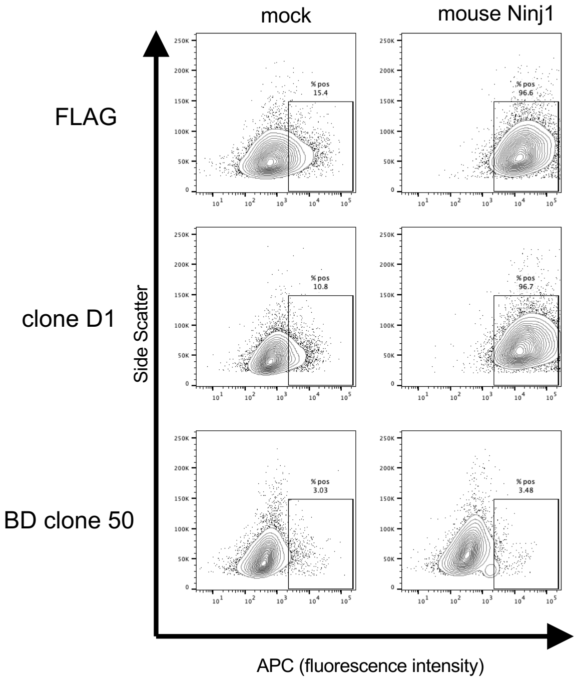

Supplement: Supplementary file 1 — This file contains Supplementary Figs. 1-4. [file 41586_2023_6191_MOESM1_ESM.pdf]
