# Supplementary material for: Inhibiting membrane rupture with NINJ1 antibodies limits tissue injury
Source: Nature. 2023 May 17;618(7967):1072–7. doi: 10.1038/s41586-023-06191-5 (PMC10307625; doi:10.1038/s41586-023-06191-5)
Supplement: Supplementary file 2 — Reporting Summary [file 41586_2023_6191_MOESM2_ESM.pdf]

nature portfolio

Corresponding author(s): Nobuhiko Kayagaki and Vishva M. Dixit

Last updated by author(s): Apr 29, 2023

Reporting Summary

Nature Portfolio wishes to improve the reproducibility of the work that we publish. This form provides structure for consistency and transparency in reporting. For further information on Nature Portfolio policies, see our Editorial Policies and the Editorial Policy Checklist.

Statistics

For all statistical analyses, confirm that the following items are present in the figure legend, table legend, main text, or Methods section.

n/a	Confirmed	
☐	☒	The exact sample size (*n*) for each experimental group/condition, given as a discrete number and unit of measurement
☐	☒	A statement on whether measurements were taken from distinct samples or whether the same sample was measured repeatedly
☐	☒	The statistical test(s) used AND whether they are one- or two-sided<br>*Only common tests should be described solely by name; describe more complex techniques in the Methods section.*
☒	☐	A description of all covariates tested
☐	☒	A description of any assumptions or corrections, such as tests of normality and adjustment for multiple comparisons
☐	☒	A full description of the statistical parameters including central tendency (e.g. means) or other basic estimates (e.g. regression coefficient) AND variation (e.g. standard deviation) or associated estimates of uncertainty (e.g. confidence intervals)
☐	☒	For null hypothesis testing, the test statistic (e.g. *F*, *t*, *r*) with confidence intervals, effect sizes, degrees of freedom and *P* value noted<br>*Give P values as exact values whenever suitable.*
☒	☐	For Bayesian analysis, information on the choice of priors and Markov chain Monte Carlo settings
☒	☐	For hierarchical and complex designs, identification of the appropriate level for tests and full reporting of outcomes
☒	☐	Estimates of effect sizes (e.g. Cohen's *d*, Pearson's *r*), indicating how they were calculated

Our web collection on statistics for biologists contains articles on many of the points above.

Software and code

Policy information about availability of computer code

Data collection	Imaging (live and fixed) data captured using MetaXpress v6.5.4.532, Incucyte S3 2019A, or Leica LAS X v3.5.7. Immunohistochemistry and histology images were acquired with Leica Application Suite v4.6.0. Flow cytometry data were acquired with BD FACSDiva software v9.1 on a BD FACSymphony. Serum ALT and AST were measured in a serum chemistry analyzer (Beckman Coulter AU480). CellTiter-Glo, LDH, HMGB1 ELISA and IL-18 ELISA data were acquired with PerkinElmer EnVision Manager 1.14.3049.1193. Size exclusion chromatography data was collected with Unicorn 7.6 (Cytiva). Negative stain data collection was done using SerialEM Version 3.9.0. For hepatic IRI studies, slides were imaged using 3DHISTECH CaseViewer v2.4 (RRID: SCR_017654; indica labs, Albuquerque, US).
Data analysis	Plots were generated with Prism 9.5.1 (GraphPad Software Inc, La Jolla, CA; RRID:SCR_002798). Imaging data were analysed and prepared using scikit-image 0.19.2. Flow cytometry data were analysed with FlowJo version 10.8.1. Incucyte data was analysed with Incucyte S3 2019A. LocusZoom v0.12 was used to generate regional association plots. For hepatic IRI studies, slides were analysed with the HALO Image Analysis Platform 3.5.3577 (RRID: SCR_018350; indica labs, Albuquerque, US). To evaluate necrosis within the hepatic I/R liver samples, the DenseNet classifier supervised machine learning algorithm (HALO Image Analysis Platform 3.5.3577) was trained to recognize necrotic tissue using the haematoxylin and eosin stain and applied to the entire sample. To quantify immune cell infiltration, neutrophils (Ly6G-positive) were counted using QuPath v0.2.1 (RRID: SCR_018257).

For manuscripts utilizing custom algorithms or software that are central to the research but not yet described in published literature, software must be made available to editors and reviewers. We strongly encourage code deposition in a community repository (e.g. GitHub). See the Nature Portfolio guidelines for submitting code & software for further information.

Data

Policy information about availability of data

All manuscripts must include a data availability statement. This statement should provide the following information, where applicable:

- Accession codes, unique identifiers, or web links for publicly available datasets
- A description of any restrictions on data availability
- For clinical datasets or third party data, please ensure that the statement adheres to our policy

The datasets generated during and/or analysed during the current study are available from the corresponding authors upon reasonable request. Source data for animal studies are provided with this paper. GWAS data was obtained from the UK Biobank study (https://doi.org/10.1038/s41588-020-00757-z).

Human research participants

Policy information about studies involving human research participants and Sex and Gender in Research.

Reporting on sex and gender	*Use the terms sex (biological attribute) and gender (shaped by social and cultural circumstances) carefully in order to avoid confusing both terms. Indicate if findings apply to only one sex or gender; describe whether sex and gender were considered in study design whether sex and/or gender was determined based on self-reporting or assigned and methods used. Provide in the source data disaggregated sex and gender data where this information has been collected, and consent has been obtained for sharing of individual-level data; provide overall numbers in this Reporting Summary. Please state if this information has not been collected. Report sex- and gender-based analyses where performed, justify reasons for lack of sex- and gender-based analysis.*
Population characteristics	*Describe the covariate-relevant population characteristics of the human research participants (e.g. age, genotypic information, past and current diagnosis and treatment categories). If you filled out the behavioural & social sciences study design questions and have nothing to add here, write "See above."*
Recruitment	*Describe how participants were recruited. Outline any potential self-selection bias or other biases that may be present and how these are likely to impact results.*
Ethics oversight	*Identify the organization(s) that approved the study protocol.*

Note that full information on the approval of the study protocol must also be provided in the manuscript.

Field-specific reporting

Please select the one below that is the best fit for your research. If you are not sure, read the appropriate sections before making your selection.

☒ Life sciences ☐ Behavioural & social sciences ☐ Ecological, evolutionary & environmental sciences

For a reference copy of the document with all sections, see nature.com/documents/nr-reporting-summary-flat.pdf

Life sciences study design

All studies must disclose on these points even when the disclosure is negative.

Sample size	Sample sizes are reported in the figure legends or the Methods section. No prior sample size calculation was performed for in vitro studies. For in vitro experiments involving BMDMs, bone marrow from at least 3 animals per genotype were analyzed for reproducibility. For in vitro experiments involving HEK 293T cells or liposome assays, three technical replicates were chosen per experiment. A minimum of three independent experiments was done for all experiments. This sample size was chosen to match previously published work by our group (Kayagaki et al, Nature 2015; Lee et al, J. Exp. Med. 2018, Kayagaki et al, Sci. Signal.) and is the norm in our field.<br><br>No prior sample size calculation was performed for in vivo studies. To account for greater variability in the in vivo studies, larger sample sizes (n=6-10) were used in the animal challenge studies (TNF+D-Gal, anti-Fas JO2, and ConA experiments) based on previous experience with the models used. These larger numbers were used to account for the greater variability between wild-type controls in these experiments. Sample sizes were chosen based on standards in the field and are sufficient based on the relatively large quantified differences between groups.
Data exclusions	For analysis of IL-18 in Fig 4f, serum from one animal was excluded in the tamoxifen-treated group due to to insufficient serum quantity. For analysis of ALT & AST serum levels in Fig 4h, one sample was excluded from the isotype control treated group due to marked icterus.
Replication	All experiments were performed independently at least twice with similar results, as described in figure legends. All attempts at replication were successful. Independent experiments and biological replicates were used to ensure reproducibility of results.
Randomization	For in vivo studies involving tamoxifen-treated animals, groups were determined by genotype rather than treatment, and therefore not

Randomization	randomized. For TNF+D-Gal, anti-Fas JO2, and ConA in vivo studies involving wt mice, animals were age- and sex- matched and randomized to group. Experimental groups were assessed in the same experiment with control groups to eliminate covariates.<br><br>For animal procedures related to hepatic ischemia-reperfusion injury mixed sex cohorts were used; animals were randomized to group and analyses blinded.<br><br>For all in vitro experiments, samples were not randomized because samples were not allocated into experimental groups.
Blinding	Imaging was performed blindly and automatically using an ImageXpress Micro Confocal or Incucyte system. Histological scoring and evaluation and serum analyses were performed blinded. For other experiments, mice and cell lines were picked and treated by the same individual, so blinding to genotype and treatment as well as during data collection and analysis was not possible.

Reporting for specific materials, systems and methods

We require information from authors about some types of materials, experimental systems and methods used in many studies. Here, indicate whether each material, system or method listed is relevant to your study. If you are not sure if a list item applies to your research, read the appropriate section before selecting a response.

Materials & experimental systems

n/a	Involved in the study
☐	☒ Antibodies
☐	☒ Eukaryotic cell lines
☒	☐ Palaeontology and archaeology
☐	☒ Animals and other organisms
☒	☐ Clinical data
☒	☐ Dual use research of concern

Methods

n/a	Involved in the study
☒	☐ ChIP-seq
☐	☒ Flow cytometry
☒	☐ MRI-based neuroimaging

Antibodies

Antibodies used	Supplementary Table 1 describes all antibodies used in this study.
Validation	Any antibody validation of commercial primary antibodies is indicated in Supplementary Table 1 and can be found on the manufacturer's websites. Non-commercial antibodies generated for this study were validated as indicated below and within the manuscript.<br><br>Clone D1-575 anti-NINJ1 antibody was validated for flow cytometry by comparing 293T cells transiently transfected with NINJ1 expression plasmids (this study, Fig1d)<br>Clone 80 anti-NINJ1 antibody was validated for immunohistochemistry and immunofluorescence by comparing tissues or cells from WT and Ninj1 KO mice (this study)<br>Clone 25 anti-NINJ1 antibody was previously validated for WB by comparing lysates from wild-type and NINJ1-/- BMDMs (Kayagaki et al . 2021 Nature 591(7848):131-136)

Eukaryotic cell lines

Policy information about cell lines and Sex and Gender in Research

Cell line source(s)	HEK293T cells (ATCC CRL-3216), Expi293F cells (Thermo Fisher Scientific, cat#A14527), BALB/3T3 clone A31 (ATCC CCL-163), CHO (Genentech), EL-4-B5 feeder cells (Roche)
Authentication	Cell lines were authenticated by short tandom repeat (STR) profiling and regular single nucleotide polymorphism (SNP) fingerprinting. STR profiles are determined for each line using the Promega PowerPlex 16 System. This is performed once and compared to external STR profiles of cell lines (when available) to determine cell line ancestry. SNP profiles are performed each time new stocks are expanded for cryopreservation. Cell line identity is verified by high-throughput SNP profiling using Fluidigm multiplexed assays. SNPs were selected based on minor allele frequency and presence on commercial genotyping platforms. SNP profiles are compared to SNP calls from available internal and external data (when available) to determine or confirm ancestry.
Mycoplasma contamination	Cells negative for mycoplasma.
Commonly misidentified lines<br>(See ICLAC register)	Not used.

Animals and other research organisms

Policy information about studies involving animals; ARRIVE guidelines recommended for reporting animal research, and Sex and Gender in Research

Laboratory animals	Mice (Mus musculus) strains including Ninj1-/- and WT littermates (Ninj1+/+) (Kayagaki et al . 2021 Nature 591(7848):131-136), and Ninj1 fl/fl Rosa26.Cre.ERT2/+ and WT littermates (Ninj1 +/+ Rosa26.Cre.ERT2/+) (this study) were maintained on a C57BL/6N genetic background.<br><br>Ninj1 fl/fl Rosa26.Cre.ERT2/+ and WT littermates (Ninj1 +/+ Rosa26.Cre.ERT2/+) were dosed with tamoxifen at 6 to 9 weeks of age.<br><br>For TNF plus D-Gal studies 8 to 14 week old female mice were used. For TNF plus D-gal studies antibody treatment studies, 8 to 14 week old age matched C57BL/6J mice were used (Jackson Labs, strain #000664).<br><br>For ConA and anti-Fas(JO2) studies, 9 to 11 week old male mice were used. For ConA and anti-Fas (JO2) studies involving antibody treatment, 9 to 11 week old age matched C57BL/6N male mice were used (Charles River Labs).<br><br>For hepatic IRI studies, mixed-sex cohorts of 6 to 10 week old C57Bl/6J wild-type animals were purchased from Jackson Laboratories (strain #000664).<br><br>Mice were housed in individually ventilated cages within animal rooms maintained on a 14:10-hour, light:dark cycle with ad libitum access to food and water. Animal rooms were temperature and humidity-controlled, between 68-79°F and 30-70% respectively, with 10 to 15 room air exchanges per hour.
Wild animals	The study did not involve wild animals.
Reporting on sex	TNF/Dgal in vivo studies were performed on 8 to 14 week old female mice. ConA and anti-Fas(JO2) studies were performed on 9 to 11 week old male mice. Hepatic IRI studies were perfomed on mixed-sex cohorts of 6 to 10 week old mice.
Field-collected samples	The study did not involve samples collected from the field.
Ethics oversight	All animal procedures were conducted under protocols approved by the Genentech Institutional Animal Care and Use Committee in an Association for Assessment and Accreditation of Laboratory Animal Care (AAALAC)-accredited facility in accordance with the Guide for the Care and Use of Laboratory Animals and applicable laws and regulations.<br><br>All animal procedures related to hepatic ischemia-reperfusion injury were conducted under protocols approved by the Animal Care Committee at The Hospital for Sick Children and in accordance with animal care regulation and policies of the Canadian Council on Animal Care.

Note that full information on the approval of the study protocol must also be provided in the manuscript.

Flow Cytometry

Plots

Confirm that:

☒ The axis labels state the marker and fluorochrome used (e.g. CD4-FITC).

☒ The axis scales are clearly visible. Include numbers along axes only for bottom left plot of group (a 'group' is an analysis of identical markers).

☒ All plots are contour plots with outliers or pseudocolor plots.

☒ A numerical value for number of cells or percentage (with statistics) is provided.

Methodology

Sample preparation	HEK293T cells (ATCC) cells were transfected with NINJ1 expression plasmids using Lipofectamine 2000 (Thermo Fisher Scientific). Cells were stained with monoclonal antibodies, followed by APC-conjugated anti-mouse IgG (Thermo Fisher Scientific) and then propidium iodide (PI; 2.5 µg/mL; BD Biosciences). Live PI– cells were analyzed in a FACSymphony (Becton 427 Dickinson).
Instrument	BD FACSymphony
Software	Data was acquired using BD FACSDiva Software v9.1, and analyzed using FlowJo 10.8.1
Cell population abundance	No sorting was performed.
Gating strategy	Dead cells that stained with PI (BD Biosciences) were excluded from analyses of cells.

☒ Tick this box to confirm that a figure exemplifying the gating strategy is provided in the Supplementary Information.
